# Evaluating reporting and process quality of publications on UNHS: a systematic review of programmes

**DOI:** 10.1186/s12887-015-0404-x

**Published:** 2015-07-22

**Authors:** Pierpaolo Mincarone, Carlo Giacomo Leo, Saverio Sabina, Daniele Costantini, Francesco Cozzolino, John B. Wong, Giuseppe Latini

**Affiliations:** Institute for Research on Population and Social Policies, National Research Council, Rome, 00185 Italy; Institute of Clinical Physiology, National Research Council, Lecce, 73100 Italy; Division of Clinical Decision Making, Department of Medicine, Tufts Medical Center, Boston, MA 02111 USA; Newborn Hearing Screening Service, Azienda USL7 Siena, Siena, 53100 Italy; Regional Health Authority of Umbria, Perugia, 06124 Italy; School of Medicine, Tufts University, Boston, MA 02111 USA; Division of Neonatology, “Perrino” Hospital, ASL Brindisi, Brindisi, 72100 Italy

**Keywords:** Neonatal screening, Process assessment (Health Care), Quality indicators, Health care, Benchmarking, Checklist

## Abstract

**Background:**

Congenital hearing loss is one of the most frequent birth defects, and Early Detection and Intervention has been found to improve language outcomes. The American Academy of Pediatrics (AAP) and the Joint Committee on Infant Hearing (JCIH) established quality of care process indicators and benchmarks for Universal Newborn Hearing Screening (UNHS). We have aggregated some of these indicators/benchmarks according to the three pillars of universality, timely detection and overreferral. When dealing with inter-comparison, relying on complete and standardised literature data becomes crucial.

The purpose of this paper is to verify whether literature data on UNHS programmes have included sufficient information to allow inter-programme comparisons according to the indicators considered.

**Methods:**

We performed a systematic search identifying UNHS studies and assessing the quality of programmes.

**Results:**

The identified 12 studies demonstrated heterogeneity in criteria for referring to further examinations during the screening phase and in identifying high-risk neonates, protocols, tests, staff, and testing environments. Our systematic review also highlighted substantial variability in reported performance data. In order to optimise the reporting of screening protocols and process performance, we propose a checklist. Another result is the difficulty in guaranteeing full respect for the criteria of universality, timely detection and overreferral.

**Conclusions:**

Standardisation in reporting UNHS experiences may also have a positive impact on inter-program comparisons, hence favouring the emergence of recognised best practices.

**Electronic supplementary material:**

The online version of this article (doi:10.1186/s12887-015-0404-x) contains supplementary material, which is available to authorized users.

## Background

The prevalence of sensorineural hearing loss ranges from 0.1 to 0.3 % for newborns [1–5] (2 to 5 % with the presence of audiological risk factors) [6]. In the absence of newborn screening, parents can only observe their infant for any inattention or unresponsiveness to sound [7, 8], often leading to delayed diagnosis of hearing loss until age 14 months on average [9]. This delay results in impaired language, learning, and speech development [10, 11], with lifelong consequences [12] including associations with increased behaviour problems, decreased psychosocial well-being, and poor adaptive skills [13–15]. Identification of hearing impairment in early childhood allows early intervention during a ‘sensitive period’ for language development [16]. More than half of babies born with hearing impairment do not have prospectively identifiable risk factors, so only universal newborn hearing screening (UNHS) programmes can identify the majority of those affected [17]. UNHS is performed via otoacoustic emission (OAE) and/or automated auditory brainstem response (aABR) testing. Neonates with positive tests are referred to audiological full evaluations for diagnosis. Children with confirmed hearing loss are managed according to early intervention strategies, which depend on the identified aetiology and may be divided into the following broad categories: audiological, medical/surgical management; educational and (re)habilitation methods; and child and family support [18].

The benefits of UNHS have been highlighted in two published systematic reviews. Nelson and colleagues [19] found that children with hearing loss identified through UNHS obtained better language outcomes at school age than those not screened, and that screened infants identified with hearing loss had significantly earlier referral, diagnosis and treatment than those not screened. Wolff and colleagues [20] determined that early identification and treatment were associated with improved long-term language development. More recently, numerous observational cohort studies [17, 21–23] have shown that early detection and intervention improve long-term reading and communication abilities when compared with no screening or late distraction hearing screening.

Differences between countries in terms of healthcare systems and the availability of resources and personnel to implement hearing screening programmes result in different approaches to implementation. Evidence from successful newborn and infant hearing screening programmes indicates several factors associated with better outcomes [24]. These include but are not limited to the following: a clearly defined and documented screening protocol; regular monitoring to ensure correct implementation of the protocol; specific training for the staff conducting the screening; quality-assurance procedures implemented to show when results are not consistent with expectations and to track what happens to all those who do not pass the screening [24].

In 1999 the American Academy of Pediatrics (AAP) *Task Force on Newborn and Infant Hearing* [9] formally supported the *Joint Committee on Infant Hearing* (JCIH) position [25] to prevent these adverse consequences through universal screening and detection of newborns with hearing loss before 3 months of age and intervention by 6 months of age, as recommended by the *National Institutes for Health* [26]. The JCIH recommendations [27, 28] form the basis for Universal Newborn Hearing Screening (UNHS) programmes developed worldwide and implemented routinely through national legislation, regional provisions or single health enterprise/hospital initiatives. Following the 1999 AAP Task Force [9], the 2000 JCIH Position Statement [27] established process and outcome performance benchmarks for Early Hearing and Detection Intervention (EHDI) programmes to evaluate UNHS progress [29] and determine programme consistency and stability [30]. Any programme not meeting these quality benchmarks should identify sources of variability and improve its processes [31]. The JCIH also suggested that hospitals and state programmes establish periodic review processes to re-evaluate benchmarks as more outcome data becomes available [27]. In 2007 [28], together with an updating of the proposed indicators for quality measure, the JCIH recommended timely and accurate monitoring of relevant quality measures as an essential practise for inter-programme comparison and continuous quality improvement.

Starting from the available statements [9, 27, 28], we have aggregated some of the already available indicators/benchmarks to evaluate the process quality of a hospital-based UNHS programme according to its three pillars of universality, timely detection and overreferral. The proposed aggregation reflects the need to measure the performance of screening programs with respect to population coverage, prompt diagnosis and consequent activation of therapeutic strategies, impact on resource consumption and stress for parents and families.

An efficient way to favour inter-programme comparison is to rely on complete and standardised literature data (avoiding, for example, the burden of contacting authors to retrieve unpublished information) and so we performed the present study to verify whether literature data reporting experiences of UNHS programmes included sufficient information to allow inter-program comparisons according to the proposed indicators.

## Methods

### Protocol

We conducted a systematic literature search to identify studies on hospital-based UNHS programmes. State-wide EHDI programmes were not taken into account.

These studies (in English) were identified by searching electronic databases, scanning reference lists of articles and consultation with experts. We excluded articles without a screening protocol description, unequivocal assignment of results to the described protocols when more than one was used, or presence of the results of a full audiological evaluation (assumed as the reference gold standard). When different publications on the same cohort were identified, only the most recent was considered, with analysis of the previous ones if relevant to retrieve missing information. Databases searched included Ovid MEDLINE (R), EMBASE, CINHAL, Cochrane Library, and Science Citation Index (Web of Science).

The search was applied, from 1990 to 2014 (last search February 5^th^, 2014) and an additional exclusion criterion was introduced, after the full-text screening phase, in order to limit the analysis to articles submitted after the publication of the first JCIH position statement (October 2000) [27] with quality indicators and benchmarks. The year 2000 was a plausible cut-off date, even with the 2007 JCIH criteria, as no additional indicator was added in the 2007 JCIH position statement with respect to the ones either suggested by the AAP in 1999 or by the JCIH in 2000. Search terms used in all databases included: child*, infant*, neonate*, newborn*, new born, paediatri*, pediatric*, hearing disorders, hearing impair*, hearing problem*, hearing defect*, hearing los*, deaf*, paracus* and dysacus*, and screen*. A fourth category of inclusion criteria was related to the study design in order to include multi-centre, observational and other types of clinical trials dealing with evaluation of programme efficacy.

Additional file 1: Appendix 1 provides the detailed search strategy. FC developed the search strings for each single database, interrogated the repositories and cleaned from duplicates. Two reviewers (PM, CGL) independently assessed abstract and full text eligibility with disagreements resolved by consensus (CGL, PM, SS, DC, JBW and GL).

This research did not involve human subjects.

The review is reported according to the *Prisma* Statement [32].

### Quality indicators and benchmarks

As suggested by the WHO, benchmarking of UNHS, i.e. evaluation of the programme against a set of quality standards, should include the minimum participation rate at screening; age at completion of the screening process; maximum referral rate; minimum participation rate and age at completion of diagnostic testing [24].

We assumed as reference indicators and benchmarks the one proposed by the AAP and JCIH whose statements are milestones recognised worldwide in the development of UNHS programs. Such indicators fulfil the WHO indications for UNHS programme monitoring [24].

We grouped the AAP [9] and JCIH [28] quality indicators and related benchmarks into three main aspects for assessment (Table 1):

**Table 1:** Quality indicators and related benchmarks assessed in our study

ID	Indicator	Benchmark	Source	Numerator	Denominator	Dimension
1	Recruitment	≥95 %	AAP^a^, 1999 and JCIH, 2007	Number of neonates that have a hearing screening test by 1 month of age	Number of neonates	Universality
	Percentage of newborns who complete screening by 1 month of age					
2	Adherence	≥70 %	AAP, 1999, JCIH, 2000	Number of neonates positive at the first screening test minus neonates who do not complete further testing (lost to follow-up)	Number of neonates positive at the first screening test	Universality
	Follow-up rate					
3	Timely definitive audiological evaluation	≥90 %	JCIH, 2007	Number of neonates undergoing definitive audiological evaluation by 3 months of age	Number of neonates undergoing a definitive audiological evaluation due to failed screening tests	Timely detection
	Percentage of newborns who complete audiological evaluation by 3 months of age					
4a	High-risk measured prevalence	2 % - 5 % (available prevalence rates [5])	Data from Scientific literature	Number of screened neonates with audiological risk factors identified with hearing loss after definitive audiological evaluation	Number of screened neonates with audiological risk factors (at net of the lost to follow-up)	Timely detection
	Observed prevalence in high-risk population					
4b	Low-risk measured prevalence	Not available (N.A.)	N.A.	Number of screened neonates without any audiological risk factor identified with hearing loss after definitive audiological evaluation	Number of screened neonates without any audiological risk factors (at net of all the lost to follow-up)	Timely detection
	Observed prevalence in low-risk population					
4c	Overall measured prevalence	0.1 % - 0.3 % (available prevalence rates [1–4])	Data from scientific literature	Number of screened neonates (with and without audiological risk factors) identified with hearing loss after definitive audiological evaluation	Number of screened neonates with or without audiological risk factors (at net of the lost to follow-up)	Timely detection
	Observed prevalence for whole population					
5	Referral rate at discharge	a) 5-20 % (for only otoemissions)	Adapted from AAP, 1999	Number of screened neonates with a positive test at the last screening test prior to hospital discharge	Number of screened neonates	Overreferral
	Referral rate before leaving the hospital					
		b) 4 % (when ABR is also used)				
6	Referral rate for definitive audiological testing after screening	<4 %	AAP, 1999 and JCIH, 2007	Number of children sent to a definitive audiological evaluation	Number of screened neonates (at net of all those lost to follow-up)	Overreferral
	Percentage of all newborn infants who fail initial screening and fail any subsequent rescreening before definitive audiological evaluation; the recommended benchmark is less than 4 %.					
7	False-positive rate	≤3 %	Adapted from AAP, 1999	Number of neonates with positive test at last screening test who have a negative definitive audiological evaluation (false positives)	Number of screened neonates without disease (false positive plus true negative^b^) at net of all those lost to follow-up.	Resource consumption
	False-positive rate related to the entire screening process					

^a^American Academy of Pediatrics

^b^When the true negatives are not available we have considered that all the negatives (i.e., all the screened minus the true positives and minus the lost to follow-up) are true negatives

1. **Universality** – measured in terms of coverage of the population in both recruitment and follow-up phases
2. **Timely detection** – evaluated according to the observed prevalence (influenced by false negatives) and the average time for diagnosis
3. **Overreferral** – estimated by referral rates in key phases of the screening programme.

### Data extraction

Pre-specified extracted information included: (1) type of study, description of programme parameters, methods for risk assessment, tests used, positivity criteria (screening threshold levels [dB] and unilateral vs. bilateral hearing loss used for the screening tests), environmental test conditions, types of personnel performing the test; (2) quality indicators. Two authors (CGL, PM) separately extracted data from each included study. Disagreements were resolved by discussion among the authors.

The authors of the selected articles were contacted whenever there was a need to obtain additional details on the reported data.

## Results

We identified 1,641 citations with eight additional citations from hand-searching personal literature files and reference lists, yielding 1,649 in all but only 1,151 non-duplicate citations. After abstract review, 90 met criteria for full text retrieval, and of these, 12 articles [33–44] met criteria for full analysis (Additional file 2: Figure S1); these include two studies [35, 38] that reported only on neonates without risk factors but were included since those were part of a UNHS programme. The following authors were contacted for additional details: Calevo [34] to specify whether the second ABR test considered was automatic or diagnostic; Guastini [37] to clarify the false-positive rate of the fourth stage considered in their programme; Kennedy [39] for clarifications on lost to follow-up; Lin [41] for clarifications on the full audiological evaluation phase. All the information provided was considered in the present work.

Table 2 describes the selected articles. The only controlled clinical trial of UNHS and the 8-year follow-up resulted in a publication also based on a preliminary report published 7 years earlier [39, 40].

**Table 2:** Study description and protocol used

ID	Source – Country – Study design	Starting year + duration in months	Criteria used for assessment of Audiological Risk	Healthcare Setting	Tests	Number of tests (before discharge; after d.; total)	Extent of Hearing Loss for screening phase	Operator performing the test	Testing environmental conditions
1	Bevilacqua M, 2010 [33] – Brasil - Hospital-based series	not exactly known, starting from 2004 to 2007 + 36 m	JCIH 2007	1 Hospital	OAE^a^	1; 1; 2	40 dB HL; unilateral	Audiologist	Non-sound-treated room (average noise < 45 dB)
2	Calevo M, 2007 [34] – Italy – multicentric Hospital-based series	2002, February + 35 m	JCIH 1994	13 Hospitals	Both^b^	1; 3; 4	50 dB SPL^b,c^; unilateral	N.R.	Sound-proof and faradized room
3	Cebulla M, 2012 [35] – Germany - Hospital-based series	2006, March + 60 m	N.R.	1 Well baby nursery - University maternity clinic	ABR^a^	1; 1; 2	35 dB nHL^d^ (aABR); unilateral	Trained physician assistants, nurses	Quiet room (stage 1); acoustically and electrically shielded room(stage 2)
4	De Capua, 2007 [36] – Italy – Multicentric hospital-based series	1998, April + 100 m	JCIH, 2000	3 hospitals	Both^e^	1; 2; 3	30 dB nHL^d^; unilateral	Technician	Silent room
5	Guastini L, 2010 [37] – Italy - Hospital-based series	2006, January + 36 m	Ad hoc	1 University Hospital	Both^b^	1; 3; 4	40 dB HL; unilateral	ENT specialists experienced in neonatal screening techniques	Sound-proof and faradised room
6	Habib H, 2005 [38] – Saudi Arabia - Hospital-based series	1996, September + 89 m	JCIH 1994	1 Hospital	OAE^a^	2; 0; 2	26 dB HL; unilateral	Technician	N.R.
7	Kennedy C, 2005 [39] - UK - Prospective cohort^f^	1993, October + 36 m	Ad hoc	4 Hospitals	Both^a^	2; 0; 2	40 dB HL; bilateral	Trained nurse	N.R.
8	Korres S, 2008 [40] – Greece - Hospital-based series	N.R. + N.R.	N.R.	1 Hospital	OAE	3; 1; 4	40 dB HL; unilateral	Audiologist	Quite room
9	Lin H, 2007 [41] – Taiwan - Retrospective cohort	a) 1998, November + 60 m;	N.R.	1 Hospital	a) OAE^a^ b) Both^a^ c) ABR^a^	a) 2–3; 0; 2–3 b) 2; 0; 2 c) 2; 0; 2	N.R.; unilateral	N.R.	N.R.
		b) 2004, February + 12 m;							
		c) 2005, March + 14 m							
10	Rohlfs AK, 2010 [42] – Germany – multicentric hospital based series	2002 August + 48 m	Ad hoc	14 birth clinics and children hospitals	Both	2; 1; 3	35 dB (aABR); unilateral	Trained nurses and physicians	N.R.
11	Tatli MM, 2007 [43] – Turkey - Prospective cohort	2002 + 18 m	Ad Hoc	1 University Hospital	OAE^a^	1; 1; 2	N.R.; unilateral	N.R.	Quite room
12	Tsuchiya H, 2006 [44] – Japan - Prospective cohort	1999, July + 64 m	N.R.	1 Hospital	ABR^a^	1; 1; 2	35 dB HL (aABR); unilateral	Technician	N.R.

^a^No differences for neonates with audiological risk were specified

^b^With both automatic and diagnostic ABR

^c^Equivalent to about 40db HL in voice frequency

^d^dB nHL = Decibel Normal Hearing

^e^Diagnostic instead of automatic ABR

^f^Data not reported in Kennedy have been gathered from Wessex [45]

Audiological risk classification was based on different criteria: JCIH recommendations [33, 34, 36, 38], and ad-hoc criteria; [37, 39, 42, 43] four studies provided no details on this point. [35, 40, 41, 44] One of the studies using JCIH criteria did not use the most recent guidelines available at the start of their recruiting period [34], and another one [36] reported using a standard that was unavailable at the start of their recruitment.

**Fig. 1:**
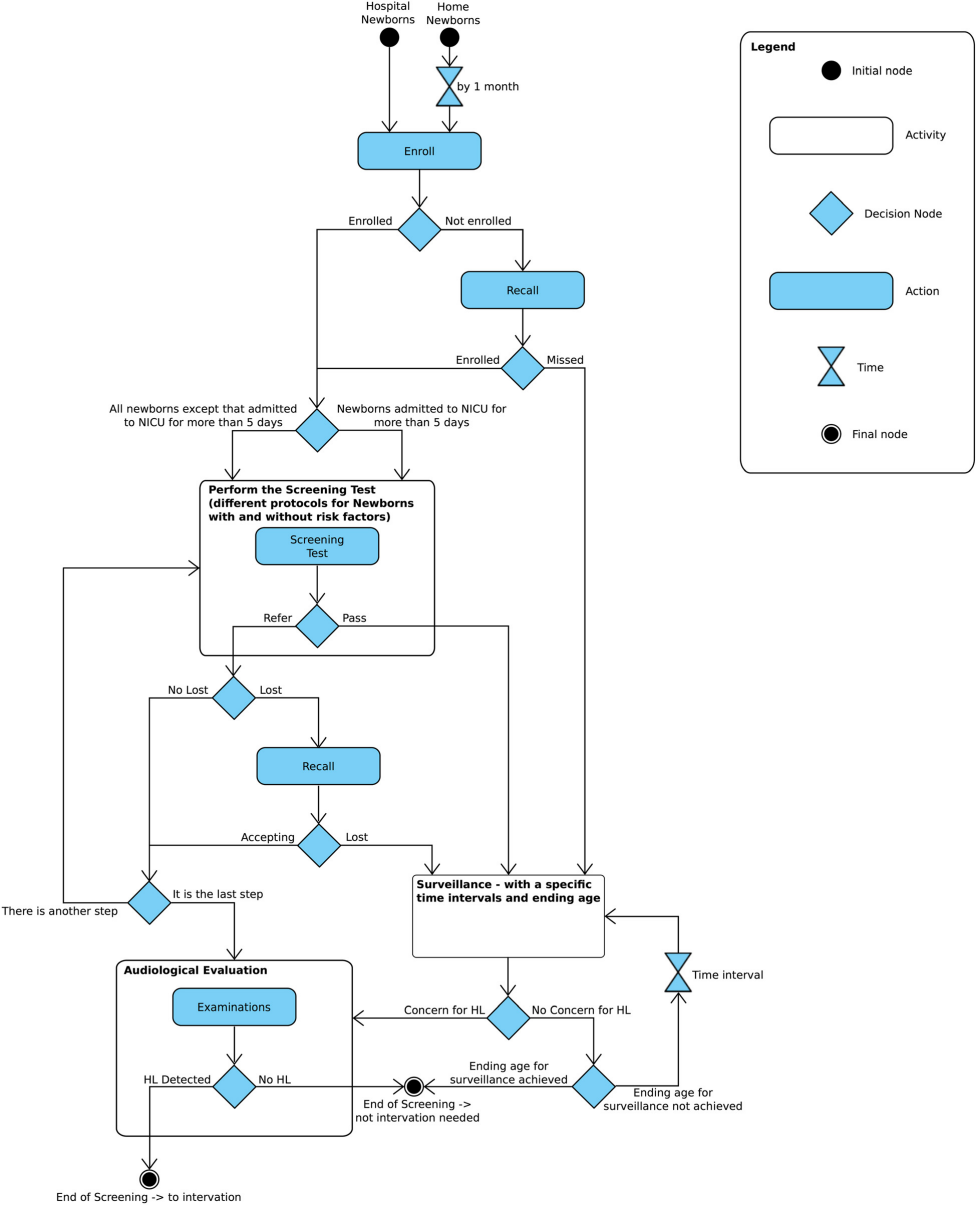
Framework for characterising the screening processes in each study

In our analysis, any examination subsequent to the first one was considered to comprise part of the follow-up phase (Fig. 1 displays the framework used for characterising the screening processes in each study). Screening examinations were carried out with one or more of the following: transient evoked otoacoustic emission (TEOAE), and/or auditory brainstem response (ABR). Although most studies use automated ABR (aABR) for screening, in three cases [34, 36, 37], diagnostic ABR (dABR), usually part of the final definitive audiological evaluation, was included within initial screening to gain additional qualitative and quantitative information about auditory nerve and brainstem pathway function. Hence, for our study we used ABR to refer to both aABR and dABR screening. In Lin and colleagues [41], dABR was performed at age 1 month, with all other gold standard examinations performed at age 3–6 months. Screening programmes involved a maximum of two [33, 35, 38, 39, 41, 43, 44], three [36, 41, 42] or four [34, 37, 40] examinations prior to definitive audiological evaluation and in six cases [33, 35, 38, 39, 41, 44] no distinction was reported for neonates with audiologic risk factors.

These 12 published studies reported on 14 screening protocols (Table 2) because during separate time frames, one study [36] reported three different protocols (only TEOAE, only ABR and both). Only otoemission was used in five protocols [33, 38, 40, 41, 43], only ABR in three [35, 41, 44] and both techniques in six [34, 36,37, 39, 41, 42]. For neonates staying in NICU for more than 5 days, five protocols [35–37, 41, 44] (the “c” protocol in Lin et al. [41]) screened with ABR as recommended by the JCIH. De Capua et al. [36] used dABR for all neonates with 2 day NICU stays, which we felt was sufficiently consistent with the 2007 JCIH recommendations. Guastini [37] used “staying in NICU for at least 48 h” as a criterion for increased audiological risk. Conversely, although Calevo et al. [34] and Rohlfs et al. [42] used ABR for all higher risk neonates, NICU stays exceeding 5 days were not considered to be a risk factor in their protocols.

The threshold for defining a positive screening test for hearing loss varied across studies: 26 [38], 30 [36], 35 [35, 42, 44] or 40 [33, 34, 37, 39, 40] dB HL (Decibel Hearing Loss) while Lin and collegues [41] and Tatli and colleagues [43] did not report any threshold. Suspected hearing loss required unilateral involvement in eleven studies [33–38, 40–44] and bilateral involvement in one [39]. The criteria for defining the presence of hearing loss at definitive audiological evaluation was identical to that used in the screening phase in all studies except in Kennedy [39]. In this study, due to the high rates of positive unilateral screening test results in neonates < 48 h old measured in the first year of the programme, the unilateral criteria for screening was changed to bilateral involvement.

Testing personnel consisted of technicians in three studies [36, 38, 44], audiologists in three [33, 37, 40], and trained physicians/assistants/nurses in three [35, 39, 42] with no specification in three [34, 41, 43] (Table 2). Testing environmental conditions were reported in seven [33–37, 40, 43] out of 12 studies (Table 2).

Results, aggregated for the three quality aspects, are summarised in Table 3 and are presented in detail in Additional file 3: Table S1, Additional file 4: Table S2, Additional file 5: Table S3 (indicators ID4a and ID4b, reported in Additional file 4: Table S2, have not been reported in Table 3). We categorised performance as *A* when the benchmark was achieved, *I* when inadequate, and *N.R.* when not reported.

**Table 3:** Overall performance indicator results

ID	Source	Universality		Timely detection		Overreferral		
		ID1	ID2	ID3	ID4c	ID5	ID6	ID7
1	Bevilacqua M, 2010 [33]	I	A	I	0.46 %	I	A	A
2	Calevo M, 2007 [34]	A	A	I	0.13 %	A	A	A
3	Cebulla M, 2012 [35]	N.R.	A	N.R.	N.R.	A	A	A
4	De Capua, 2007 [36]	I	A	A	0.18 %	A	A	A
5	Guastini L, 2010 [37]	N.R.	A	I	0,07 %	A	A	A
6	Habib H, 2005 [38]	I	A	N.R.	N.R.	N.R.	A	A
7	Kennedy C, 2005 [39]	I	A	I	0.10 %	A	A	A
8	Korres S, 2008 [40]	N.R.	I	N.R.	N.R.	A	A	A
9	Lin H, 2007 [41]	N.R.	a) A	N.R.	Protocols	A	Protocols	Protocols
			b) I		a) 0.46 %		a) I	a) I
			c) A		b) 0.25 %		b) A	b) A
					c) 0.42 %		c) A	c) A
10	Rohlfs AK, 2010 [42]	I	I	I	0,20 %	A	A	A
11	Tatli MM, 2007 [43]	N.R.	A	N.R.	0.42 %	N.R.	A	A
12	Tsuchiya H, 2006 [44]	I	A	N.R.	N.R.	N.R.	A	A

For *Universality* (Additional file 3: Table S1), one study [34] achieved the benchmark of 95 % screened in the first month of life (ID1). Out of the six [33, 36, 38, 39, 42, 44] that failed to meet that standard, one study had a 66.5 % [44] average screening rate (due to a low 38.4 % performance in the first reported year) with the others ranging from 83.2 % [39] to 93.2 % [36]. Five studies [35, 37, 40, 41, 43] could not be evaluated for this benchmark. For follow-up (ID2), ten [33–39, 41] ^(only protocols with either OAE or ABR),^[43, 44] achieved the 70 % benchmark; three [40, 41] ^(only protocol with OAE and ABR),^ [42] reported 27.1 % [40] to 65.1 % [42] follow-up [39].

For *Timely detection* (Additional file 4: Table S2), audiological evaluation completion by 3 months of age (ID3) could only be assessed with certainty in one study [36]. Among the remaining eleven, five [33, 34, 37, 39, 42] reported the age at diagnosis using criteria that differed from the one recommended by the JCIH (90^th^ percentile diagnosed by 3 months of age). Six studies [35, 38, 40, 41, 43, 44] did not report results on this issue. Overall measured prevalence – i.e., ID4c – (Additional file 4: Table S2) varied from 0.07 % [37] to 0.46 % [33, 41] with observed prevalence rates of 1.1 % [37] to 4.89 % [34] for neonates at higher risk (ID4a) [34, 36, 37], and 0.04 % [37] to 0.68 % [35] (ID4b) for neonates without audiological risk factors [34–38, 40, 44]. ID4a and ID4b are not reported in Table 3 but only in Additional file 4: Table S2.

For *Overreferral* (Additional file 5: Table S3), our assessment was based on the overall study population (i.e., neonates either with or without risk factors). It is important, when reading the indicators, to consider the mix of the population with respect to risk factors. In fact, neonates classified as higher risk comprised 1.4 % to 11.2 % [33, 34, 36, 37, 39] of those screened and can determine a change in the value of the indicators due to the different risk of hearing loss within the two populations. Eight studies [34–37, 39–42] achieved the referral after hospital discharge benchmark (ID5). This criterion was not met in one study [33] and was not evaluable in three [38, 43, 44].

All the studies achieved the referral rate benchmark for definitive audiological evaluation for all newborn infants who failed initial screening and any subsequent rescreening – ID6 – (in the case of Lin et al. [41] the benchmark was not achieved in the protocol using only OAE). Exactly the same result is verified for the false-positive rate benchmark (ID7).

## Discussion

The purpose of this study was to verify whether literature data on hospital-based UNHS programmes included sufficient information to allow inter-programme comparisons according to the considered indicators, taken from available internationally recognised position statements [9, 27, 28], and aggregated according to the pillars of *universality*, *timely detection* and *overreferral*.

We found that not all studies reported all the data necessary for calculating the complete proposed set of quality indicators, and that when comparing available data on indicators with corresponding benchmarks, the full achievement of all the recommended targets is an open challenge.

Additional considerations may be made from the above-reported results.

We found substantial heterogeneity in the literature data in the criteria for hearing loss detection (bilateral or unilateral; threshold – in this case in line with the finding of another systematic review [46]), the criteria for identifying high-risk neonates, the screening tests used, the personnel performing the tests, and the environment in which the tests were carried out.

Two of the benchmarks considered for *Overreferral* (ID5 and ID6 in Additional file 5: Table S3) suggest the proportion of neonates that should be referred after discharge and of those sent for definitive audiological evaluation after screening. In clinical practise, achieving these benchmarks may be adversely affected if an institution has a high proportion of higher-risk neonates in its population. Adjustment for case-mix by having separate benchmarks for the higher- and average-risk populations would resolve this potential issue. Moreover, these benchmarks may be affected in cases where neonates with audiological risk factors are directed to subsequent steps or to the full audiological evaluation even when passing a screening test.

Our study confirms the presence of another important bias in evaluating programme performances, due to the difficulty in identifying the false negative cases. In fact, this requires excellent cooperation between health organisations and the ability to evaluate whether any possible hearing loss identified at a later age has an acquired, late-onset, progressive aetiology or is a true false negative.

Finally, incomplete follow-up, besides potentially missing some neonates with hearing loss, may also under- or over-estimate programme performance by affecting the age at diagnosis, prevalence, percentage of newborns eligible for definitive audiological evaluation, and false positive rate. As reported in Kemper and Downs [47], although in the United States UNHS has been universally adopted, a key challenge has been identified in assuring that screening is consistently administered with good follow-up and that those identified with hearing impairment receive effective intervention. Assuring follow-up after screening is especially difficult [48]. In fact, in a US survey [49] only 62 % of all newborns with positive screening tests completed definitive diagnostic evaluation; of these, only 52 % were evaluated by 3 months of age as recommended by the JCIH. Loss to follow-up at all stages of the EHDI process also continues to be a serious concern for the World Health Organization (WHO) [24], which emphasises the importance of monitoring and implementing all phases of screening (responsibilities, training, information campaign, procedures of quality assurance). As averred by the AAP [50] and endorsed by the JCIH [28], EHDI should include a *surveillance phase* in which infants up to 30 months of age undergo monitoring for auditory skills, middle-ear status, and developmental milestones. This can lead to earlier detection of hearing loss in infants who had been lost to follow-up, as well as identifying false negatives missed at UNHS.

Our contribution focuses on UNHS programmes. For this reason we referred only to the subset of the indicators developed by the AAP and JCIH, which since their statements are focused on EHDI, also allow assessment of the quality of the diagnostic, treatment and follow-up processes. A more extensive application of our approach for the full monitoring of EHDI programmes should consider this limitation.

Moreover, it must be considered that the referred benchmark values are based on expert opinion and that it is not clear whether achieving or not achieving them directly correlates with a threshold where benefits outweigh any harm of screening or vice versa. An un-reflected adoption of such benchmarks then seems inadequate.

Another limitation is the restriction to articles written in English. However, many authors from non-English-speaking countries often publish in English-language journals; in the present review all but one [39] considered studies originating from non-English-speaking countries.

An additional limitation derives from the fact that the 12 retrieved studies are probably not representative of the existing UNHS programmes worldwide. This depends on the presence of results not published in peer-reviewed journals indexed in bibliographic databases, but issued as reports in the *gray literature*.

## Conclusions

As reported in the 2007 JCIH Position Statement [28], regular measurement of performance and routine monitoring of indicators are recommended for inter-programme comparison and continuous quality improvement. Frequent assessment of quality permits prompt recognition and correction of any unstable component of the EHDI process [51]. Our systematic review of UNHS studies highlights substantial variability in programme design and in reported performance data. In order to optimise reporting of screening protocols and process performance we propose a checklist (Table 4). In developing this list, we have relied on the investigative approach used in our study: description of the protocol and analysis of the aspects of quality indicators (*Universality*, *Timely detection*, and *Overreferral*). Future studies should address the following critical areas: assessment of long-term outcomes of neonates with negative screening tests, causes for and interventions to reduce patients lost to follow-up, the standardisation of recommended quality indicators, and the definition of evidence-based benchmarks.

**Table 4:** Reporting checklist

Protocol Section/item	Item #	Description	Reported on page #/line #
Configuration of hearing loss	1	Identify the target of the screening as unilateral or bilateral.	
Severity scale	2	Provide the scale used to classify the degree of hearing loss (e.g., normal, mild, moderate, and severe)	
Threshold dB for hearing loss	3	Provide the dB hearing loss threshold and rationale for that choice	
Criteria used for assessing audiological risks	4	Specify the criteria used to define higher audiological risks (e.g., the JCIH 2007, admission to NICU, or other criteria)	
Protocol for NICUneonates	5	Specify protocol used to screen NICU neonates admitted for more than 5 days (JCIH recommends automatic auditory brainstem response -- aABR)	
Protocol for otherneonates	6	Specify protocol used to screen all the other categories of neonates	
Testing environment conditions	7	Describe the environment in which the test is performed (e.g., NICU, quiet room, mother’s bed)	
Definitive audiological evaluation tests	8	Describe the tests used to perform the definitive audiological examination as the gold standard for diagnosing hearing loss in neonates who have positive screening tests	
Actions for missed and lost to follow up	9	Describe the actions performed to re-contact newborns who were missed or lost to follow-up during one or more screening exam steps	
Developmental surveillance and monitoring	10a	Describe any continued hearing surveillance to detect hearing loss in all children less than 30 months old and all methods used to detect missed cases of hearing loss during neonatal screening, e.g., due to late onset of hearing loss or false-negative test results during screening tests	
	10b	Describe additional methods (beyond those in 10a) used to identify hearing loss that was undetected (i.e., false negatives) during neonatal screening, (e.g., information from services providing hearing aids)	
	10c	Describe subsequent developmental monitoring for special populations of children with hearing loss, including those with minimal and mild bilateral hearing loss, unilateral hearing loss, and neural hearing loss	
Communication	11	Describe all methods used to inform parents about hearing loss screening and results before, during and after the screening	
Health personnel	12	Specify the health personnel performing the screening and their role in each exam (e.g., physicians for programme coordination and communication with parents, nurses for newborn wellness screening and data management, audiologist for hearing examination)	
Quality Indicators			
A) Universality			
Recruitment	13	Percentage of newborns who complete screening by 1 month of age	
		Numerator: Number of neonates that have a hearing screening test by 1 month of age	
		Denominator: Number of neonates	
Adherence	14	Follow-up rate	
		Numerator: Number of neonates positive at the first screening test minus neonates who do not complete further testing (lost to follow-up)	
		Denominator: Number of neonates positive at the first screening test	
B) Timely detection			
Timely definitive audiological evaluation	15	Percentage of newborns with definitive audiological evaluation by 3 months of age	
		Numerator: Number of neonates undergoing definitive audiological evaluation by 3 months of age	
		Denominator: Number of neonates undergoing a definitive audiological evaluation	
High-risk measured prevalence	16	Observed prevalence in high-risk population	
		Numerator: Number of screened neonates with audiological risk factors identified with hearing loss after definitive audiological evaluation	
		Denominator: Number of screened neonates with audiological risk factors (at net of the lost to follow-up)	
Low-risk measured prevalence	17	Observed prevalence in low-risk population	
		Numerator: Number of screened neonates without any audiological risk factor identified with hearing loss after definitive audiological evaluation	
		Denominator: Number of screened neonates without any audiological risk factor (at net of those lost to follow-up)	
Overall measured prevalence	18	Observed prevalence for whole population	
		Numerator: Number of screened neonates (with and without audiological risk factors) identified with hearing loss after definitive audiological evaluation	
		Denominator: Number of screened neonates with or without audiological risk factors (at net of those lost to follow-up)	
C) Overreferral			
Referral rate at discharge	19	Referral rate before leaving the hospital	
		Numerator: Number of screened neonates with a positive test at the last screening test prior to hospital discharge	
		Denominator: Number of screened neonates	
Referral rate for definitive audiological Testing after screening	20	Percentage of all newborn infants who fail initial screening and fail all subsequent re-screening before comprehensive audiological evaluation	
		Numerator: Number of children completing definitive audiological evaluation	
		Denominator: Number of screened neonates (at net of the lost to follow-up)	
False-positive rate	21	False-positive rate related to the entire screening process	
		Numerator: Number of neonates who have a negative definitive audiological evaluation (false-positive screening tests)	
		Denominator: Number of screened neonates without disease (false positives plus true negatives or equivalently, number of screened neonates minus the number of neonates found to truly have hearing loss after definitive audiological evaluation and minus the number of neonates with hearing loss found negative at the screening) at net of those lost to follow-up	

Another result inferable from an initial analysis of available data is the difficulty in guaranteeing full respect for the criteria of universality, timely detection and overreferral. Standardisation in reporting UNHS experiences may also have a positive impact on inter-programme comparisons, hence favouring the emergence of recognised best practises.

## Additional files

Additional file 1:
**SEARCH STRATEGY – Medline via OVID.** The document reports the search strategy used to retrieve abstracts from Medline/OVID. Additional file 2: Figure S1. PRISMA Flow Diagram. The figure reports a summary of screened articles. Additional file 3: Table S1. Universality Performance Indicators. The table describes the detailed evaluations of indicators ID1 and ID2. Additional file 4: Table S2. Timely detection Performance Indicators. The table describes the detailed evaluations of indicators ID3, ID4a, ID4b, ID4c. Additional file 5: Table S3. Overeferral Performance Indicators. The table describes the detailed evaluations of indicators ID5, ID6 and ID7.

## Abbreviations

aABR: Automatic auditory brainstem response
AAP: American academy of pediatrics
ABR: Auditory brainstem response
dABR: Diagnostic auditory brainstem response
EHDI: Early hearing detection and intervention
JCIH: Joint committee on infant hearing
NICU: Neonatal intensive care unit
OAE: Otoacoustic emission
UNHS: Universal newborn hearing screening
