# Supplementary material for: Evaluating reporting and process quality of publications on UNHS: a systematic review of programmes
Source: BMC Pediatr. 2015 Jul 22;15:86. doi: 10.1186/s12887-015-0404-x (PMC4511235; doi:10.1186/s12887-015-0404-x)
Supplement: Additional file 1: — SEARCH STRATEGY – Medline via OVID. The document reports the search strategy used to retrieve abstracts from Medline/OVID. [file 12887_2015_404_MOESM1_ESM.pdf]

APPENDIX 1 - SEARCH STRATEGY – Medline via OVID

1. exp child/
2. exp infant/
3. child$.ti,ab,hw.
4. neonat$.ti,ab,hw.
5. infant$.ti,ab,hw.
6. (newborn$ or (new adj1 born)).ti,ab,hw.
7. (paediatri$ or pediatri$).ti,ab,hw.
8. 1 or 2 or 3 or 4 or 5 or 6 or 7
9. exp hearing disorders/
10. exp hearing impaired persons/
11. (hearing adj (disorder$ or los$ or impair$)).ti,ab,hw.
12. hearing.ti,ab,hw.
13. (deaf$4 or hearing NEAR loss$2 or hearing NEAR disorder$2 or paracus$2 or dysacus$2 or hearing NEAR impair$4 or hearing NEAR problem$1 or hearing NEAR defect$3).ti,ab.
14. 9 or 10 or 11 or 12 or 13
15. exp Mass Screening/
16. exp Neonatal Screening/
17. screen$3.ti,ab,hw.
18. 15 or 16 or 17
19. exp clinical trial/
20. exp research design/
21. exp treatment outcome/
22. exp double-blind method/

23. exp single-blind method/

24. exp comparative study/

25. ((single or double or triple) adj3 blind$3).ti,ab,hw.

26. random$.ti,ab,hw.

27. controlled clinical trial.pt.

28. practice guideline.pt.

29. clinical trial.pt.

30. (clinical adj trial$1).ti,ab,hw.

31. (control$3 adj trial$1).ti,ab,hw.

32. randomi#ed controlled trial.pt.

33. pla#ebo$.ti,ab,hw.

34. clinical trial.mp.

35. random$.mp.

36. tu.xs.

37. 19 or 20 or 21 or 22 or 23 or 25 or 26 or 27 or 28 or 29 or 30 or 31 or 32 or 33 or 34 or 35 or 36

38. 8 and 14 and 18 and 37

39. limit 38 to (humans and yr="1990 -Current")
