# Supplementary figures and images for: Evaluating reporting and process quality of publications on UNHS: a systematic review of programmes
Source: BMC Pediatr. 2015 Jul 22;15:86. doi: 10.1186/s12887-015-0404-x (PMC4511235; doi:10.1186/s12887-015-0404-x)

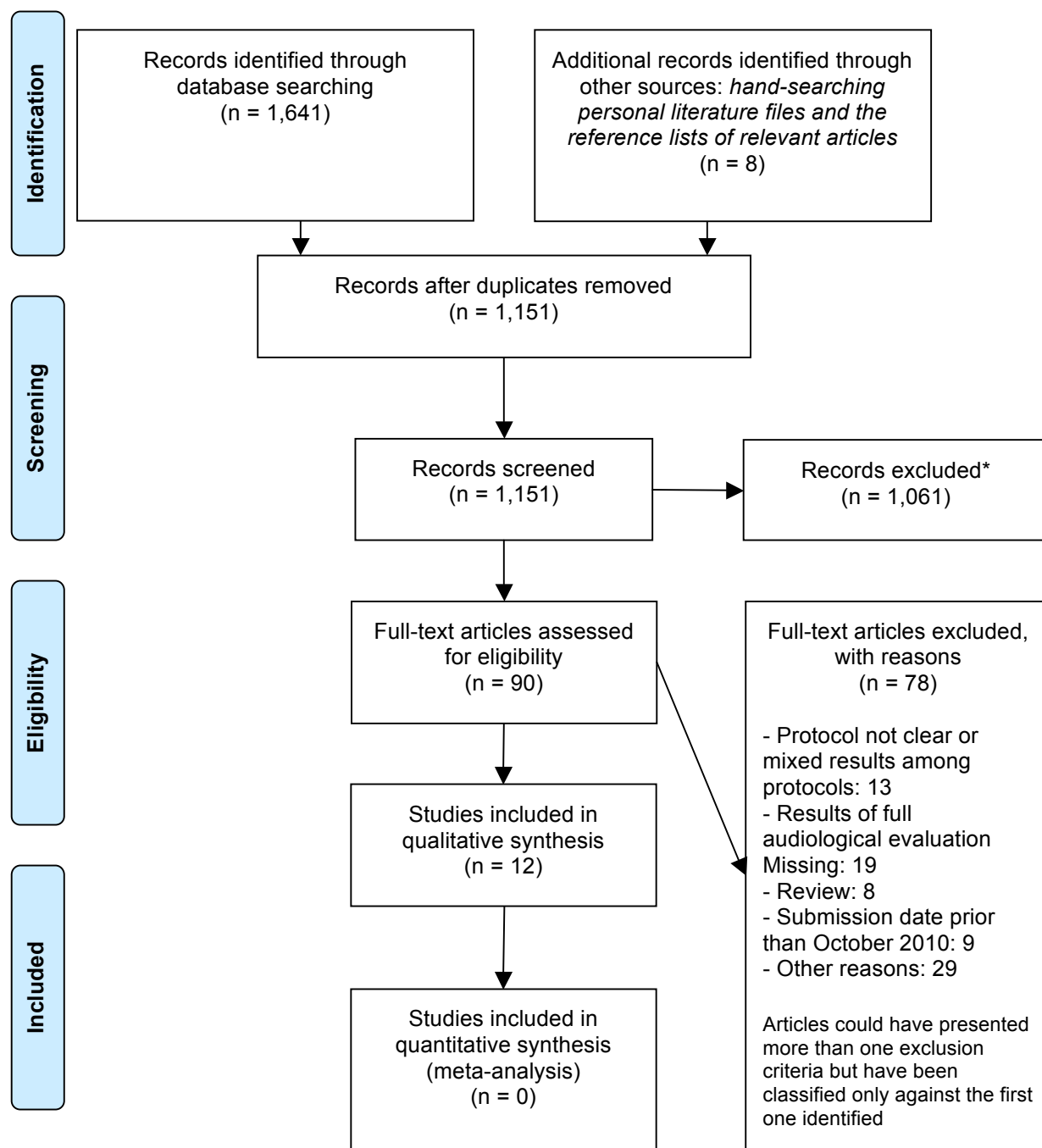

**Fig. S1.** Flow diagram of eligible studies

Supplement: Additional file 2: Figure S1. — PRISMA Flow Diagram. The figure reports a summary of screened articles. [file 12887_2015_404_MOESM2_ESM.pdf]
