# Supplementary material for: Evaluating reporting and process quality of publications on UNHS: a systematic review of programmes
Source: BMC Pediatr. 2015 Jul 22;15:86. doi: 10.1186/s12887-015-0404-x (PMC4511235; doi:10.1186/s12887-015-0404-x)
Supplement: Additional file 3: Table S1. — Universality Performance Indicators. The table describes the detailed evaluations of indicators ID1 and ID2. [file 12887_2015_404_MOESM3_ESM.pdf]

Table S1. Universality Performance Indicators

ID	Source	I1: Recruitment	I1 ≥95% [A[a], I[b], N.R.[c]]	I2: Adherence	I2 ≥ 70% [A, I, N.R.]
1	Bevilacqua M, 2010[33]	11,466 / 12,667 (90.5%)	I	1 – 519 / 2,546 (79.6%)	A
2	Calevo M, 2007[34]	32,296 / 32,502 (99.4%)	A	1 – 240 / 3,474 (94.5%)	A
3	Cebulla M, 2012[35]	6,868 / N.R. (N.R.)	N.R.	1 – 2 / 261 (99.2%)	A
4	De Capua, 2007[36]	19,700 / 21,125 (93.2%)	I	1 – 255 / 2,893 (91.2%)	A
5	Guastini L, 2010[37]	8,671 / N.R. (N.R.)	N.R.	1 – 42 / 694 (93.9%)	A
6	Habib H, 2005[38]	11,986 / N.R. (91.7%)	I (high-risk newborns not recruited)	1 – 0 / 300 (100.0%)	A
7	Kennedy C, 2005[39]	21,303 / 25,609 (83.2%)	I	1 – 0 / N.R. (100%)	A
8	Korres S, 2008[40]	76,560 / N.R. (N.R.)	N.R.	1 - 1,230 / 1,688 (27.1%)	I
9	Lin H, 2007[41]	Three Protocols used (see Table 2):<br>a) 18,260 / N.R. (N.R.);<br>b) 3,540 / N.R. (N.R.);<br>c) 3,788 / N.R. (N.R.)	N.R.	a) 1 – 196 / 1,055 (81.4%);<br>b) 1 – 17 / 57 (70.2%);<br>c) 1 – 3 / 31 (90.3%)	a) A<br>b) I<br>c) A
10	Rohlfs AK, 2010[42]	(59,010 + 1,772[d]) / 65,466 (92.8%)	I	1 - 701 / 1.963 (65.1%)	I

[a] A = Achieved
[b] I = Inadequate (i.e., Benchmark not achieved)
[c] N.R. = Not Reported / Benchmark not evaluable
[d] 59,010 refers to neonates screened before discharge and 1,772 to neonates not screened primarily but screened at the second stage within 1 month of age

ID	Source	I1: Recruitment	I1 ≥95% [A[a], I[b], N.R.[c]]	I2: Adherence	I2 ≥ 70% [A, I, N.R.]
11	Tatli MM, 2007[43]	711 / N.R. (N.R.)	N.R.	1 - 6 / 28 (78.6%)	A
12	Tsuchiya H, 2006[44]	1999: 554 / 1,444 (38.4%);<br>2004: 1,968 / 2,170 (90.7%);<br>From, 1999 to 2004: 8,979 / 13,494 (66.5%)	I<br>I<br>I	From 1999 to 2004: 1 - 1 / 37 (97.3%)	A
