# Supplementary material for: Evaluating reporting and process quality of publications on UNHS: a systematic review of programmes
Source: BMC Pediatr. 2015 Jul 22;15:86. doi: 10.1186/s12887-015-0404-x (PMC4511235; doi:10.1186/s12887-015-0404-x)
Supplement: Additional file 4: Table S2. — Timely detection Performance Indicators. The table describes the detailed evaluations of indicators ID3, ID4a, ID4b, ID4c. [file 12887_2015_404_MOESM4_ESM.pdf]

Table S2. Timely detection Performance Indicators

ID	Source	I3: Timely definitive audiological evaluation[a]	% of newborns who complete definitive audiological evaluation by 3 months of age ≥90% [A, I, N.R.]	*I4:* High-risk measured prevalence [b]	*I5:* Low-risk measured prevalence	*I6:* Overall measured prevalence [c]
1	Bevilacqua M, 2010[33]	age at diagnosis varied from 1 month to 13 months: 6 children with Hearing Loss within 3 months; two < 1y; three >1y but not older than 2 years	I	N.R.	N.R.	51 / (11,466 – 519) (0.46%)
2	Calevo M, 2007[34]	median age<br>- well-born: 6.7 months;<br>- at risk: 6.9 months	I	22 / (510 – 60) (4.89%)	19 / (31,796 – 180) (0.06%)	41 / (32,258 – 240) (0.13%)
3	Cebulla M, 2012[35]	Mean age for well born: 2,9 months	N.R.	N.R.	0.68% (95% CI: 0.49–0.88%).	N.R.
4	De Capua, 2007[36]	All infants diagnosed with congenital Hearing Loss had completed diagnoses by 3 months of age	A	24 / 1,334[d] (1.8%)	11 / 18,356d (0.1%)	35 / (19,700 – 255) (0.18%)
5	Guastini L, 2010[37]	Reported in the protocol: 6 months of age	I	3 / 264 (1.1%)	3 / (8,407 – 42) (0.04%)	6 / (8,671 – 42) (0,07%)
6	Habib H, 2005[38]	Average confirmation age of congenital hearing loss was 5.5 months	N.R.	Not considered	22 / 11,986 (0.18%)	N.R. / N.R. (N.R.)

[a] When no data were available on the age of diagnosis, N.R. has been reported; when diagnosis age was inferable from the protocol we have added “protocols report that …”; for all studies where the reporting format for age of diagnosis has been reported differently from that suggested by the JCIH (e.g. mean, median, 75% percentile instead of the recommended 90% percentile), we have used text to describe the age at diagnosis.
[b] Prevalence rates reported in literature: 2-5% (reported in Table 1)
[c] Prevalence rates reported in literature: 0.1-0.3% (reported in Table 1)
[d] The 255 lost to follow-up neonates are not classifiable against risk factors and have not been removed

ID	Source	I3: Timely definitive audiological evaluation[a]	% of newborns who complete definitive audiological evaluation by 3 months of age ≥90% [A, I, N.R.]	*I4:* High-risk measured prevalence [b]	*I5:* Low-risk measured prevalence	*I6:* Overall measured prevalence [c]
7	Kennedy C, 2005[39]	16/27 (59%) by 10 months vs 10/26 (38%) without UNHS	I	N.R. / 1,724 (N.R.)	NA / 19,555 (N.R.)	22 / 21,279 (0.10%)
8	Korres S, 2008[40]	N.R.	N.R.	N.R. / N.R. (N.R.)	56 / (76,560 – 1,230) (0.07%)	N.R. / N.R. (N.R.)
9	Lin H, 2007[41]	N.R.	N.R.	N.R.	N.R.	Three protocols (see Table 2):<br>a) 83 / (18,260 – 196) (0.46%);<br>b) 9 / (3,540 – 17) (0.25%);<br>c) 16 / (3,788 – 3) (0.42%)
10	Rohlfs AK, 2010[42]	Median age (months):<br>- well born: 3,1<br>- NICU: 4,2	I	N.R. / N.R. (N.R.)	N.R. / N.R. (N.R.)	118 / (60,782 – 701) (0,20%)
11	Tatli MM, 2007[43]	N.R.	N.R.	2 / N.R. (N.R.)	1 / N.R. (N.R.)	3 / (711 – 6) (0.42%)
12	Tsuchiya H, 2006[44]	N.R.	N.R.	N.R.	24 / (8,979 – 1) (0.27%)	N.R. / N.R. (N.R.)
