# Supplementary material for: Evaluating reporting and process quality of publications on UNHS: a systematic review of programmes
Source: BMC Pediatr. 2015 Jul 22;15:86. doi: 10.1186/s12887-015-0404-x (PMC4511235; doi:10.1186/s12887-015-0404-x)
Supplement: Additional file 5: Table S3. — Overeferral Performance Indicators. The table describes the detailed evaluations of indicators ID5, ID6 and ID7. [file 12887_2015_404_MOESM5_ESM.pdf]

Table S3. Overeferral Performance Indicators

ID	Source	# with hearing loss risk factors / # screened (%)	I5: Referral rate after discharge (% of screened)	I5 within 5-20% (for OAE) or ≤4% (for ABR or OAE+ABR) [A, I, N.R.]	I6: Referral rate for definitive audiological evaluation after screening	I6 <4% [A, I, N.R.]	I7: False Positive Rate	I7 ≤3% [A, I, N.R.]
1	Bevilacqua M, 2010[33]	1,284 / 11,466 (11.2%)	2,546 / 11,466 (22.2%)	**I** – OAE	382 / (11,466 – 519) (3.5%)	A	312 / (10,893 – 519) (3.0%)	A
2	Calevo M, 2007[34]	462 / 32,306 (1.4%)	- without audiological risk factor: 3,012 / 31,796 (9.5%);<br>- at higher audiological risk: 510 / 510 (100.0%)<br>- globally: 3,522 / 32,306 (10.9%)	A – OAE	a) without audiological risk factor: 19 / (31,796 – 180) (0.06%)[a]<br>b) at higher audiological risk: 22 / 462 (4.8%)<br>Globally: 41 / 32,258 (0.1%)	A	0,28% (reported)	A
3	Cebulla M, 2012[35]	N.R. / 6868 (N.R.%)	Well baby newborn: 261 / 6,868 (3.8%)	**A -** aABR	188 / (6,868 - 2) (2.7%)	A	141 / (6,819 – 2) (2.1%)	A
4	De Capua, 2007[36]	1,344 / 19,700 (6.8%)	≤2,893 / 19,700 (14.7%)[b]	**A** – OAE	35 / (19,700 – 255) (0.2%)	A	0 / (19,665 – 255) (0.0%)	A
5	Guastini L, 2010[37]	264 / 8,671 (3.0%)	- without audiological risk factor: 430 / 8,407 (5.1%);<br>- at higher audiological risk: 264 / 264 (100.0%)<br>- globally: 694 / 8,671 (8,0%)	**A** – OAE	6 / (8,671 – 42) (0.07%)	A	0 / (8,665 – 42) (0.0%)	A

[a] 3 neonates with transient Hearing Loss resolved by month 12 have been included

[b] 2,893 is the sum of 1,549 positive at first TOAE and 1,344 High Risk (among which a number of neonates classified as *TOAE refer* were included); even for the worst case of no high risk neonate without OAE the benchmark would be achieved.

ID	Source	# with hearing loss risk factors / # screened (%)	I5: Referral rate after discharge (% of screened)	I5 within 5-20% (for OAE) or ≤4% (for ABR or OAE+ABR) [A, I, N.R.]	I6: Referral rate for definitive audiological evaluation after screening	I6 <4% [A, I, N.R.]	I7: False Positive Rate	I7 ≤3% [A, I, N.R.]
6	Habib H, 2005[38]	0 / 11,986 (0%)	without audiological risk factor: 300 / 11,986 (2.5%)	**N.R.** – OAE	300 / 11,986 (2.5%)	A	278 / 11,986 (2.3%)	A
7	Kennedy C, 2005[39]	1,724 / 21,279 (8.1%)	N.R. / N.R. (2.5% at the start of the program. to 1.6% at a later stage)	**A** - OAE/ABR	N.R. / N.R. (2.5% at the start of the program. to 1.6% at a later stage)	A	319 / 21,279 (1.5%)	A
8	Korres S, 2008[40]	N.R. / 76,560 (N.R.)	1,688 / 76,560 (2.2%)	**A** - OAE	404 / (76,560 – 1,230) (0.5%)	A	99 / (75,330 – 1,230) (0.1%)	A
9	Lin H, 2007[41]	Three Protocols used (see Tab 2): a) N.R. / 18,260 (N.R.); b) N.R. / 3,540 (N.R.); c) N.R. / 3,788 (N.R.);	a) 5.8% (2-3 tests); b) 1.6% (2 tests); c) 0.8% (2 tests)	a) **A** - OAE; b) **A** – OAE/ABR; c) **A** - ABR	Three protocols used (see Table 2): a) 1,055 / (18,260 – 196) (5.8%); b) 57 / (3,540 – 17) (1.6%); c) 31 / (3,788 – 3) (0.8%)	a) I; b) A; c) A	a) 776 / (18,064 – 196) (4.3%); b) 31 / (3,523 – 17) (0.9%); c) 12 / (3,785 – 3) (0.3%)	a) I; b) A; c) A
10	Rohlfs AK, 2010[42]	N.R. / 60.782 (N.R.)	1.963 / 60,782 (3,2%)	**A** - ABR	217 / (60,782 - 701) (0.4%)	A	33 / (60,664 – 701) (0.05%)	A
11	Tatli MM, 2007[43]	N.R. / 711 (N.R.)[c]	N.R. / N.R. (N.R.)	**N.R.** - OAE	5 / (711 – 6) (0.7%)	A	0.3% reported by the authors	A

[c] Authors report that 236 out of 711 (33.2%) are from NICU

ID	Source	# with hearing loss risk factors / # screened (%)	I5: Referral rate after discharge (% of screened)	I5 within 5-20% (for OAE) or ≤4% (for ABR or OAE+ABR) [A, I, N.R.]	I6: Referral rate for definitive audiological evaluation after screening	I6 <4% [A, I, N.R.]	I7: False Positive Rate	I7 ≤3% [A, I, N.R.]
12	Tsuchiya H, 2006[44]	N.R. / 8,979 (high risk neonates needing intensive care not included)	37 / 8,979 (0.4%)	**N.R.** - ABR	31 / (8,979 – 1) (0.3%)	A	6 / (8,978 – 1) (0.07%)	A
